# Evaluation of adaptability and stability for iron, zinc and protein content in cowpea genotypes using GGE biplot approach

**DOI:** 10.1016/j.heliyon.2022.e11832

**Published:** 2022-11-30

**Authors:** Maurício dos Santos Araújo, Walter Frazão Lelis de Aragão, Samíria Pinheiro dos Santos, Thaise Kessiane Teixeira Freitas, Verônica da Costa Saraiva, Kaesel Jackson Damasceno-Silva, Luiz Antônio dos Santos Dias, Maurisrael de Moura Rocha

**Affiliations:** a Universidade Federal de Viçosa, Departamento de Agronomia, 36570-900, Viçosa, Minas Gerais, Brazil; b Universidade Federal do Piauí, Departamento de Fitotecnia, 64049-550, Teresina, Piauí, Brazil; c Universidade Federal do Piauí, Departamento do Centro de Ciências da Saúde, 64049-550, Teresina, Piauí, Brazil; d Empresa Brasileira de Pesquisa Agropecuária, (Embrapa Meio-Norte), 64008-780, Teresina, Piauí, Brazil

**Keywords:** *Vigna unguiculata*, Genotype-by-environment interaction, GGE biplot Analysis, Nutritional quality

## Abstract

Cowpea is a widely cultivated crop in the world. Biofortification strategies aim to reduce mineral and protein deficiencies, especially among the poorest people. The aim of this study was to estimate adaptability and stability of cowpea genotypes for iron, zinc and protein contents, through GGE biplot analysis. Twenty cowpea genotypes were evaluated in the municipalities of Piauí Monsenhor Hipólito, Pio IX and São Miguel do Tapuio, under rainfed conditions. The experimental design was a randomized block design with four replications. The traits evaluated were grain yield, iron, zinc and protein contents in dry grains. Iron (Fe) and zinc (Zn) were determined by flame atomic absorption spectrophotometer, and protein contents by Kjeldahl methods. Adaptability and stability were evaluated by GGE biplot analyses. The means of the experiments were 1,209.1 kg ha^−1^, 51.1 mg kg^−1^, 46.8 mg kg^−1^ and 24.3% for grain yield, Fe, Zn and protein contents, respectively. The joint analysis of variance showed significant difference (*p* < 0.05) for the effect of interaction genotypes by environments for Fe, Zn and protein contents. The lines G6 and G8 were the most promising for grain yield, mineral and protein content through adaptability and stability by GGE biplot approach.

## 1 Introduction

Cowpea [*Vigna unguiculata* (L.) Walp.] is a crop of African origin, cultivated in Africa, Asia, United States of America, and the Latin America, due to its wide genetic variability (Issoufa et al., 2020; Santos et al., 2020). Brazil is considered the third largest producer of cowpea in the world with the harvested area, production, and yield of 1,354.0 kg ha^−1^, 716.9 tonnes, and 528 kg ha^−1^, respectively, in the 2021/2022 year (Conab, 2021). Higher grain yield is obtained in the Northeast and North regions of Brazil due to cowpea adaptation in arid and semi-arid conditions (Okoth et al., 2017). The crop is a source of essential amino acids (Carvalho et al., 2012), proteins (Weng et al., 2019), minerals (Gondwe et al., 2019), vitamins (Dakora; Belane 2019), polyphenolic compounds and antioxidants, being an important food of the human diet (Khang et al., 2016; Moreira-Araújo et al., 2017; Alidu et al., 2020).

According to the World Health Organization (WHO) nutritional deficiency is a global health problem. Two billion people worldwide consume insufficient levels of micronutrients, protein and vitamins in daily diet (Who, 2022). Women of reproductive age and children under five are more vulnerable to nutritional deficiency. Malnutrition is caused by lack of micronutrients and insufficient intake of Fe, Zn and vitamin A (Carvalho et al., 2015). The Fe and Zn participate in major biological functions such as in the composition of hemoglobin (Rouault, 2015), catalytic cofactor in various biochemical reactions (Hohenberger et al., 2012), nucleic acid metabolism, gene expression, growth and differentiation (Black et al., 2013; Roohani et al., 2013; Koop et al., 2018; Tardy et al., 2020). The lack of these minerals can cause severe damage to human homeostasis (Lonergan; Skaar, 2019).

Biofortification aims to increase mineral, protein and vitamin contents in a crop (López-Morales et al., 2020). This strategy is promising to minimize human malnutrition (Kihara et al., 2020). HarvestPlus seeks to nutritionally improve agricultural crops to improve human nutrition. This global strategy occurs through partnerships between research institutions and executive agencies. It has allowed the development of cultivars with high vitamin and mineral contents, acting as a global leader. Thus, the institution has collaborated with the genetic improvement of the cowpea, especially in the northeast region of Brazil where it is an important ingredient in basic nutrition (HarvestPlus 2021). The increased bioavailability of nutrients in crops can be achieved through the genetic or agronomic way (Ullah et al., 2020). The genetic way applies breeding methods to increase mineral, protein and vitamin content (Bouis; Saltzman 2017). The agronomic approach uses fertilizer application on soil or leaf area to complement the micronutrient contents of main crops (Cakmak; Kutman 2017; Garg et al., 2018).

The main problem in recommending biofortified cultivar is the existence of genotype-by-environment interaction (GEI) (Naik et al., 2020). The GEI can mask the genotypic value due to the environmental influence (Cruz et al., 2014). The better way to exploit GEI is to perform adaptability and stability analyses. The adaptability and stability parameters are basically evaluated by regression models (Finlay; Wilkinson 1963; Eberhart; Russell 1966), segmented regression (Cruz et al., 1989), nonparametric analysis (Lin; Binns 1988), and multiplicative analyses such as GGE (Genotype main effect plus genotype-by-environment interaction) biplot (Yan et al., 2000; Yan, 2014) and AMMI (additive main effect and multiplicative interaction) (Gauch, 2006). The AMMI approach combines the analysis of variance and the principal components analysis to adjust, respectively, the main effects (genotypes and environments) and the effects of the GEI. However, GGE biplot groups the additive effect of genotype with the multiplicative effect of GEI, and subjects these to principal components analysis (Yan et al., 2000; Yan, 2014).

Adaptability and stability studies for yield in cowpea is common in literature (Mohammed; Amsalu 2018; Abreu et al., 2019; Baraki et al., 2020), but for Fe, Zn and protein contents in the grain are scarces, due to be the main target traits in biofortification programmes for cowpea (Silva; Santos 2017; Cardona-Ayala et al., 2021), mainly using GGE biplot analysis (Oliveira et al., 2017). The selection of cowpea genotypes efficient in translocating these nutrients from the root to the grain and capable of tolerating water stress is important for the development of biofortified cultivars with adaptations to these edaphoclimatic conditions, enabling the recommendation to farmers. The aim of this study was to evaluate the adaptability and stability for Fe, Zn and protein contents in the grain of cowpea genotypes using the GGE biplot approach.

## 2 Material and methods

### 2.1 Genetic materials

The genetic material was composed of 17 inbred lines and three commercial cultivars (Table 1). These 20 genotypes are regular materials from the Cowpea Breeding Program of Embrapa Meio-Norte, in Teresina, Piauí state, Brazil, with genetic potential for biofortification of mineral and protein contents. However, they have already passed through preliminary, intermediate, and are in the Value for Cultivation and Use (VCU) trials. The trials were conducted in the 2019/2020 crop year. This last step aimed to evaluate grain yield stability in different environments before cultivar recommendation. These experiments are carried out in a collaborative network and are necessary for the registration of a cultivar with the Ministério da Agricultura, Pecuária e Abastecimento in Brazil.

**Table 1:** Description of cowpea genotypes evaluated in the semiarid zone of northeastern Brazil.

Code	Genotype	Genealogy	Commercial^1^ subclass
G1	MNC11–1013E-33	MNC01–510F x Pingo-de-Ouro-1–2	ML
G2	MNC11–1013E-16	MNC01–510F x Pingo-de-Ouro-1–2	EG
G3	MNC11–1013E-15	MNC01–510F x Pingo-de-Ouro-1–2	ML
G4	MNC11–1013E-35	MNC01–510F x Pingo-de-Ouro-1–2	EG
G5	MNC11–1018E-17	MNC02677F-2–2 x MNC01–631–20–5 x Pingo de Ouro-1–2	EG
G6	MNC11–1019E-8	MNC01–631F-11 x Canapuzinho-2 x MNC02–677F-2–1	ML
G7	MNC11–1019E-12	MNC01–631F-11 x Canapuzinho-2 x MNC02–677F-2–1	ML
G8	MNC11–1019E-46	MNC01–631F-11 x Canapuzinho-2 x MNC02–677F-2–1	ML
G9	MNC11–1020E-16	MNC02–689F-11 x MNC02–677F-2–1	ML
G10	MNC11–1022E-58	MNC02–689F-11 x MNC01–631F-20–5 x MNC99–510F-16–1	ML
G11	MNC11–1024E-1	MNC02–689F-11 x MNC99–510F-16–1 x Pingo-de-Ouro-1–2	ML
G12	MNC11–1026E-15	MNC02–689F-11 x MNC01–631F-11 x Canapuzinho-2	ML
G13	MNC11–1026E-19	MNC02–689F-11 x MNC01–631F-11 x Canapuzinho-2	ML
G14	MNC11–1031E-5	MNC02–689F-11 x MNC02–680F-1–2	EG
G15	MNC11–1031E-11	MNC02–689F-11 x MNC02–680F-1–2	ML
G16	MNC11–1034E-2	MNC01–631F-20–5 x Pingo-de-Ouro-1–2 x MNC02–761F-2	ML
G17	MNC11–1052E-3	Bico de Ouro-1–2–1 x MNC01–631F-20–5 x MNC99–510F-16–1	CN
G18	BRS Pajeú	CNCx405–17F x TE94–268–3D	ML
G19	BRS Marataoã	Seridó x TVx 1836–013J	ML
G20	BRS Rouxinol	TE86–75–57E x TEx1–69E	EG

1 Mulate (ML), Evergreen (EG), and Canapu (CN).

### 2.2 Experimental design

The field trials were evaluated at three sites in the semi-arid zone of the state of Piauí, Brazil (Table 2). The environments present a tropical climate with dry winter, following the Köppen-Geiger classification (Alvares et al., 2013). The soil of all environments evaluated in this study was Red-Yellow Latosol with sandy texture.

**Table 2:** Description of the environments evaluated in the semi-arid zone of Piauí, Brazil.

Environment	ID	Location		Altitude (m)	Annual rainfall mean
		Latitude	Longitude		
Monsenhor Hipólito	MS	07° 00' 06"	41° 01' 46"	262	800–1400
Pio IX	PIX	06° 50'15"	40° 34' 45"	494	600–700
São Miguel do Tapuio	SMT	05° 29' 46"	41° 18' 46"	272	800–1400

The crop management followed Freire-Filho et al. (2012). Soil preparation in both trials consisted of plowing followed by harrowing. Crop treatments consisted of applying a pre-emergence herbicide based on s-metolachlor (1L ha^−1^) and manual weeding between 20 and 30 days, representatively for weed control. After planting, insect control was performed at the beginning of the cycle (aphids), at flowering (thrips) and at the beginning of fruit formation (caterpillars and bed bugs), using insecticides based on dimethoate (1L ha^−1^) and thiamethoxam (100 g ha^−1^). The harvest was done manually around 70 days after planting.

The field trials were conducted in a randomized complete block design with four replications. The genotypes were arranged in four 5.0 m rows. The plants were spaced at 0.50 m × 0.25 m, with a useful area of 5 m^2^ formed by the two central rows, which were used to measure the traits. The Red-Yellow Podzolic was common in the three locations evaluated. The tested environments were chosen due to being part of the cowpea evaluation network for the selection of a biofortified cultivar for the semi-arid zone of Piauí.

### 2.3 Traits

Grain yield (GY), zinc content (Zn), iron content (Fe), and protein content (Prot).

#### 2.3.1 Grain yield

The GY was obtained by weighing the grains obtained in the useful area of the plot, and then converting it to kilograms per hectare (kg ha^−1^), considering spacing and plot length (Ongom et al., 2021).

#### 2.3.2 Laboratory analyses

The nutritional analyses were carried out in the Bromatology Laboratory at the Empresa Brasileira de Pesquisa Agropecuária, in the city of Teresina, Piauí, Brazil. Samples of grains of the genotypes randomly taken in the useful area of three repetitions of the multi-trials were used. The nutritional traits evaluated in the study were Prot, Fe and Zn contents. Two hundred grams of grains per genotype/environment were ground in a zirconium ball mill (MM 200, Germany) to obtain the flour. Analyzes were accomplished following the methods of the Association of Official Analytical Chemists (Aoac, 2005). For each replication, the analyzes were performed in triplicate.

##### 2.3.2.1 Fe and Zn quantification

Fe and Zn contents analyses were performed with the nitroperchloric digestion of 0.2 g flour obtained of each genotype. A total of 20 mL distilled water was added to the digested extract. The solution was stirred (Vortex 0–3000 RPMs, USA), and samples were read using a flame atomic absorption spectrophotometer (iCE 3000 Series, Massachusetts).

##### 2.3.2.2 Protein quantification

We use the Micro-Kjeldahl method to quantify the protein content of dry grains in cowpea (Aoac, 2005). Two hundred milligrams (mg) of flour were weighed. The protein digestion tube (Kjeldahl) was used to allocate the sample. The sample was weighted with 2 g of the catalytic solution (96.5% K_2_SO_4_ and 3.5% CuSO_4_) and 5 mL of sulfuric acid. Then, 10 mL of distilled water were added for distillation of Nitrogen/protein distiller (Te-0363-Agroads, São Paulo). Then we inserted 15 mL of 50% NaOH into the tube.

The quantification of nitrogen was determined by Eq. (1):

(1) $$Total\;nitrogen=\frac{VHAT\;x\;F\;x\;0.14}{W}$$

Where: VHAT = is the volume total (mL) of HCl that was used in the titration for each sample; F = is the correction factor; W = sample weight.

The total protein content was corrected for dry matter, which was obtained after drying at 106 °C for 12h. The conversion factor of nitrogen in protein was 6.25 (Mariotti et al., 2008). The final measurement of the protein content (%) in the sample was obtained by Eq. (2).

(2) PC=TNx6.25

Where: Protein content (PC); total nitrogen (TN).

### 2.4 Data analysis

Initially, we performed the individual analysis of variance for each environment. Then, joint analysis was performed with the three trials. The means were grouped by the Scott−Knott test (*p*<0.05). The effect of genotype was considered as fixed and environment, as random (Cruz et al., 2014). Individual analysis of variance for GY followed the statistical model of Eq. (3):

(3) $$Y_{ij}=\mu +G_{i}+B_{J}+\varepsilon_{\dot{ij}}$$

Where: $Y_{ij}$^:^ is the observed value for the response variable obtained for the i-th genotype in the j-th block^;^ μ^:^ overall mean^;^ $G_{i}$: the effect of the genotype i; $B_{J}$: the effect of the block j; $\varepsilon_{\dot{ij}}$: is the error associated with the observation ij.

The joint analysis of variance for GY followed the statistical of Eq. (4):

(4) $$Y_{ijk}=\mu +{\left(B/E\right)}_{jk}+E_{J}+G_{i}+{GE}_{ij}+\varepsilon_{\dot{ij}k}$$

Where: $Y_{ijk}$: is the observed value of genotype i in the environment j and block k; μ: overall mean; ${\left(B/E\right)}_{jk}$: is the interaction of block k within the environment j; $E_{J}$: is the effect of environment j; $G_{i}$: is the effect of the genotype i; ${GE}_{ij}$: is the interaction between genotype i and environment j; $\varepsilon_{\dot{ij}k}$: is the error associated with the observation i jk.

For analysis of iron, zinc, and protein content in the grains, we used completely randomized design. In Eq. (5) the individual analysis of variance is reported:

(5) $$Y_{ij}=\mu +G_{i}+\varepsilon_{\dot{ij}}$$

Where: $Y_{ij}$: the observation of the genotype i in the repetition j; μ: overall mean of observations; $G_{i}$: the effect of the genotype i; $\varepsilon_{\dot{ij}}$: the error associated with the observation i j.

Eq. (6) describes the model of the joint analysis for iron, zinc and protein content:

(6) $$Y_{ij}=\mu +G_{i}+E_{j}+{GE}_{ij}+\varepsilon_{\dot{ij}}$$

Where: $Y_{ij}$: is the observed value of the genotype i in the environment j; μ: is the overall mean; $G_{i}$: is the effect of the genotype i; $E_{j}$: is the effect of the environment j; ${GE}_{ij}$: is the interaction between genotype i and environment j; $\varepsilon_{\dot{ij}}$: is the error associated with the observation ij.

The grouping of means for nutritional traits in each environment was performed by the Scott and Knott (1974). The evaluation of the adaptability and stability was performed by the GGE biplot analysis, proposed by Yan (2014), according to Eq. (7).

(7) $${\bar{Y}}_{ij}-\mu -E_{j}=G_{i}+GE_{ij}$$

Where: ${\bar{Y}}_{ij}$: the genotypic value of genotype i in environment j; μ: is the overall mean; $E_{j}\text{:}$ is the main effect of the environment j; $G_{i}$: is the main effect of genotype i; ${GE}_{ij}\text{:}$ is the interaction between genotype i and environment j;

For the GGE biplot approach, the effects of G and GxE are the most important, and there is the joint presence of both multiplicative terms, as described in Eq. (8):

(8) $$Y_{ij}-\mu -\beta_{j}=g_{i1}e_{i1+}g_{i2}e_{i2+}\epsilon_{ij}$$

Where: $Y_{ij}\text{:}$ is the expected yield of genotype i in the environment j; μ: the overall mean; $\beta_{j}\text{:}$ is the main effect of the environment j; $g_{i_{1}}$ e $e_{i_{1}}$: is the main scores of genotype i and environment j, respectively; $g_{i_{2}}$ and $e_{i_{2}}$: is the secondary scores for genotype i and environment j, respectively; $\varepsilon_{ij}\text{:}$ the error not explained by both effects. The graph is obtained by simple dispersion of $g_{i_{1}}$ and $g_{i_{2}}$ for genotypes and, $e_{i_{1}}$ and $e_{i_{2}}$ for environments, by the decomposition of the singular value Eq. (9):

(9) $$Y_{ij}-{\bar{Y}}_{j}=\lambda_{1}\varepsilon_{i_{1}}\rho_{j_{1}}+\lambda_{2}{\;\varepsilon }_{i_{2}}\rho_{j_{2}}+\varepsilon_{ij}$$

Where: $\lambda_{1}$ and $\lambda_{2}$: is the highest eigenvalues of the first (PC1) and second (PC2) principal components, respectively; $\varepsilon_{i_{1}}$ and $\varepsilon_{i_{2}}$: the eigenvalues of genotype i for PC1 and PC2, respectively; $\rho_{j_{1}}$ and $\rho_{j_{2}}$: the eigenvalues of environment j for PC1 and PC2, respectively (Yan; Tinker 2006). All analyses were performed in R software (R Core Team 2022), using the packages “*agricolae*” (Mendiburu, 2021) and “*Metan*” (Olivoto; Lúcio, 2020).

## *3* Results

### 3.1 Analyses of variance

The mean square of the environment effect was significant (*p* < 0.01) by analysis of variance for trait GY. However, we did not identify any significant difference for the effects of G and GEI. The average GY per environment is presented in Table 3. In addition, mean values of Fe, Zn, Prot, and GY for each genotype per trial are presented in Table 4.

**Table 3:** Analysis of joint variance for cowpea yield in the semiarid zone of Piaui, Northeastern Brazil.

Source of variation	DF	Mean squares
		GY (kg ha^−1^)
Block/Environment	9	277668.5
Environment (E)	2	5063382.32∗∗
Genotype (G)	19	145237.51^ns^
G x E	38	89398.59^ns^
Error	171	92861.26
Total	239	
Overall mean		1209.13
CV (%)		25.44

∗Significant at *p* < 0.05, ∗∗*p* < 0.01 (Snedecor’s F-test). DF:degree of freedom. CV:coefficient of variation. G x E: genotype-by-environmental interaction; GY: grain yield.

**Table 4:** Mean values of the 20 cowpea genotypes for iron zinc and protein contents cultivated in three environments of the Piauí semi-arid zone of Northeastern Brazil.

ID	Fe^1^ (mg kg^−1^)			Zn (mg kg^−1^)			Prot (%)			GY (kg ha^−1^)		
	MSH	PIX	SMT	MSH	PIX	SMT	MSH	PIX	SMT	MSH	PIX	SMT
G1	47.79c	42.93b	58.48b	23.60f	25.87a	24.81c	43.64b	47.30a	44.29a	808.80a	1416.85a	1466.90a
G2	56.28b	40.57b	61.20b	24.90c	24.37c	24.83c	50.16a	49.37a	48.29a	875.20a	1246.53a	1402.65a
G3	52.77b	37.07b	68.44a	25.20b	25.94a	24.40d	46.54b	43.58b	53.31a	884.550a	1604.68a	1572.60a
G4	46.39c	36.78b	76.81a	24.03e	24.98b	23.76f	46.92b	46.49a	47.16a	905.93a	1660.23a	1629.40a
G5	45.51c	54.20a	57.37b	22.53i	26.22a	23.41g	47.35b	42.11b	51.23a	903.70a	1387.83a	994.60b
G6	66.05a	45.32b	64.02b	24.86c	25.53b	25.54b	53.68a	49.69a	46.38a	1043.95a	1533.43a	1157.00b
G7	56.26b	40.63b	73.12a	22.85h	25.35b	23.29g	50.82a	42.10b	47.72a	1025.55a	1252.70a	1428.00a
G8	62.47a	56.48a	73.78a	26.07a	25.98a	24.24e	52.37a	48.87a	51.88a	951.18a	1307.35a	1253.20b
G9	63.45a	41.50b	70.46a	21.32j	22.74d	23.42g	47.09b	45.93a	46.59a	976.05a	1060.18a	1391.10a
G10	57.82b	45.11b	64.70b	25.20b	25.23b	23.22g	49.96a	48.44a	47.75a	1022.95a	1327.43a	1098.75b
G11	50.51c	44.83b	51.46c	23.26g	23.79c	23.16g	44.10b	43.82b	59.30a	962.03a	1388.03a	1465.50a
G12	65.09a	46.57b	58.88b	22.91h	24.18c	23.76f	53.65a	46.97a	46.07a	805.08a	1340.45a	1303.30b
G13	42.74c	58.69a	38.23d	23.45f	25.14b	24.84c	51.83a	44.81a	45.71a	1028.90a	1215.90a	1386.30a
G14	42.36c	53.89a	31.68d	23.58f	24.55c	23.94f	52.04a	41.56b	48.56a	743.38a	1206.35a	1474.60a
G15	44.63c	49.03a	55.72b	21.15j	24.11c	22.80h	49.87b	40.76b	48.42a	1015.85a	1415.35a	1261.55b
G16	43.21c	52.99a	43.30c	24.46d	26.07a	25.95a	55.00a	44.39a	38.74a	605.88a	968.23a	1074.05b
G17	42.16c	53.05a	28.80d	23.19g	24.19c	23.83f	47.15b	45.52a	44.95a	1011.68a	1388.60a	1460.90a
G18	38.46c	43.70b	48.90c	26.08a	23.90c	24.89c	43.57b	36.47b	44.51a	753.95a	522.18a	1283.00b
G19	41.30c	44.81b	51.90c	24.71c	25.98a	25.48b	51.52a	40.58b	45.34a	782.40a	1557.78a	1283.45b
G20	35.30c	48.29a	48.90c	25.24b	24.80c	25.05c	47.28b	41.14b	42.13a	735.70a	1127.35a	1402.80a

Mean values followed by the same letter belong to the same group by the Scott and Knott test (*p* < 0.05); ^1^Fe: iron content; Zn: zinc content; Prot: protein content; GY: grain yield; MSH: Monsenhor Hipólito; PIX: Pio IX; SMT: São Miguel do Tapuio

The effect of E and GEI were significant (*p* < 0.01) for all nutritional traits. However, only protein content exhibited variability for genotype effect by the pooled ANOVA. The overall average for minerals and protein is presented in Table 5.

**Table 5:** Analysis of joint variance for minerals (iron and zinc) and protein in cowpea grains in the semiarid zone of Piaui, Northeastern Brazil.

Source of variation	DF	Mean squares		
		Fe (mg kg^−1^)	Zn (mg kg^−1^)	Prot (%)
Genotypes (G)	19	350.17^ns^	41.66^ns^	7.03∗∗
Environments (E)	2	1205.55∗∗	305.45∗∗	16.32∗∗
GxE	38	292.60∗∗	41.62∗∗	1.99∗∗
Error	120	46.30	21.74	0.09
Total	179			
Overall mean		51.10	46.87	24.37
CV (%)		13.32	9.94	1.22

∗Significant at *p* < 0.05, ∗∗*p* < 0.01 (Snedecor’s F-test); Fe: iron content; Zn: zinc content; Prot: protein content; CV: coefficient of variation ​; ns: not significant.

### 3.2 GGE biplot analysis

**Figure 1:**
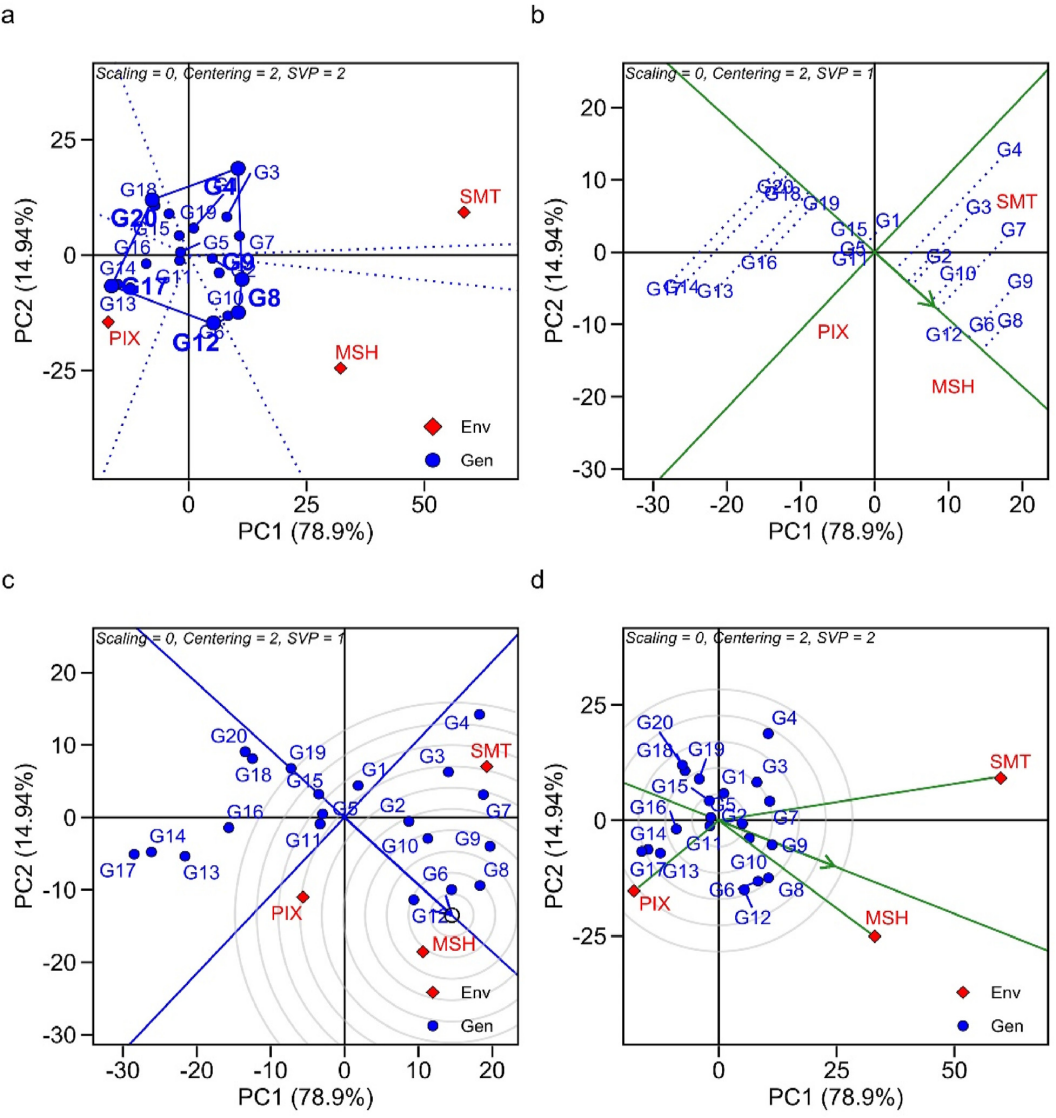
GGE biplot analysis for adaptability and stability in terms of iron content in 20 cowpea genotypes, evaluated in three environments of the Piauí State semi-arid, Brazil; (a) Which-Won-Where; (b) Average *vs.* Stability; (c) Ideal genotype; (d) Discriminant and representative environment.

**Figure 2:**
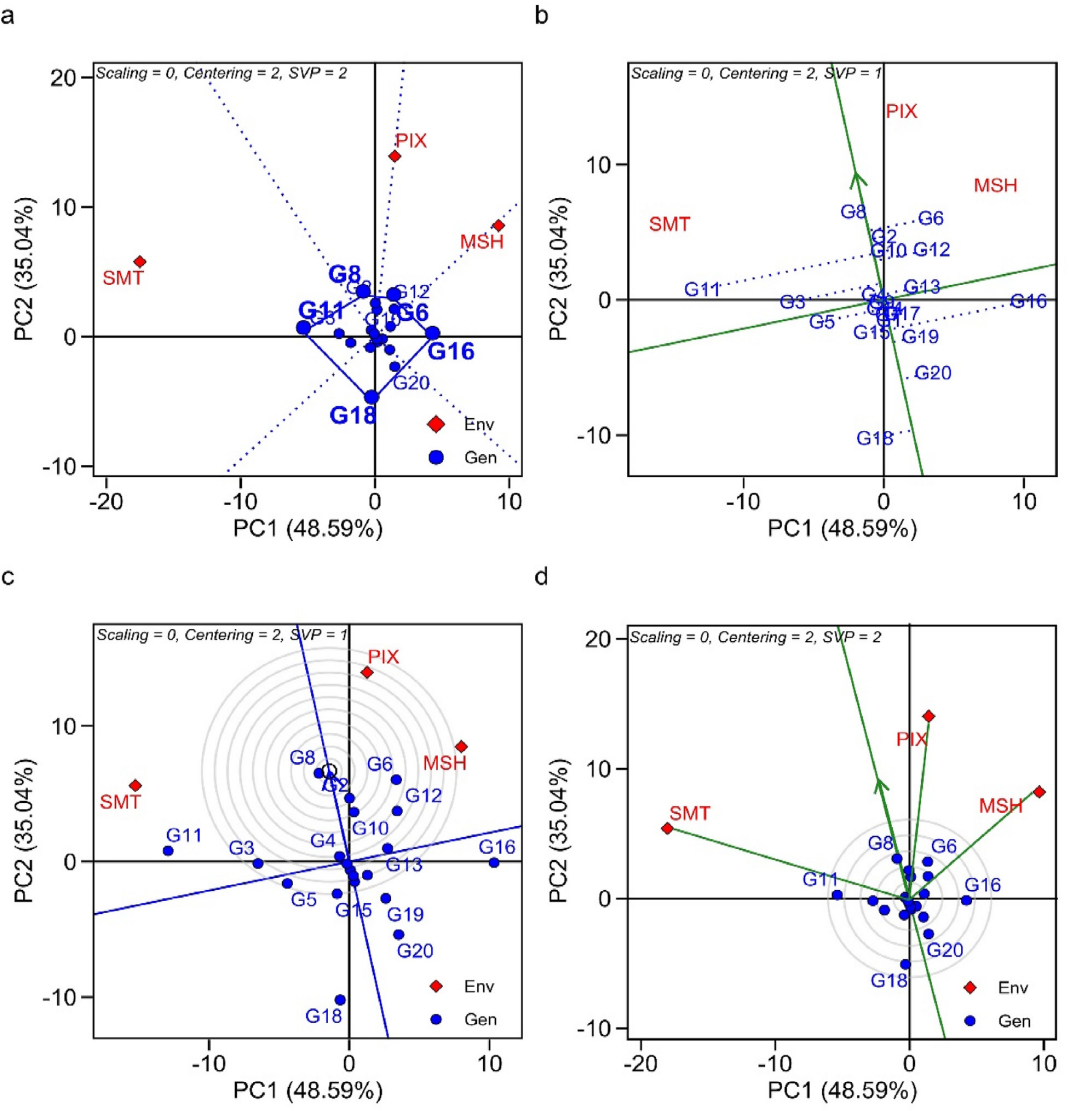
GGE biplot analysis for adaptability and stability regarding zinc content in 20 cowpea genotypes, evaluated in three environments of the Piauí state semi-arid zone, Brazil; (a) Which-Won-Where; (b) Average *vs.* Stability; (c) Ideal genotype; (d) Discriminant and representative environment.

**Figure 3:**
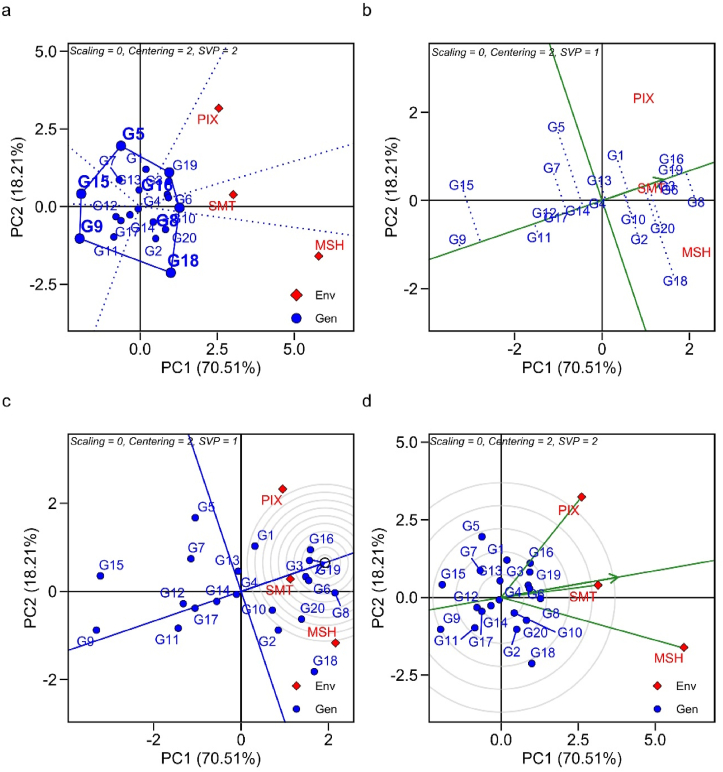
GGE biplot analysis for adaptability and stability regarding protein content in 20 cowpea genotypes, evaluated in three environments of the Piauí state semi-arid zone, Brazil; (a) Which-Won-Where; (b) Average *vs.* Stability; (c) Ideal genotype; (d) Discriminant and representative environment.

Principal component analysis (PCA) integrates the GGE biplot approach, as it highlights through adaptability and stability the importance of superior genotypes in the evaluated environments. The two principal components together explained 93.84% of the total variation in the sum of squares (PC1 78.9%; PC2 14.94%), for the trait iron content, for exemple (Figure 1). The graph ''which-won-where'' is divided by vectors arising from origin of the biplot (0.0) forming the sectors. The genotypes that are farthest from the origin of the biplot represent those with the highest GY and adaptation (Figures 1a, 2a and 3a).

The average environment coordinate (AEC) or average environment axes (AEA) is represented by two arrows antiparallel to the origin of the biplot. This highlights the greater effect of GEI and a lower stability. Therefore, the arrows differentiate the genotypes that are above or below the average. The 'average *vs*. stability' biplot, often stated as AEC and singular value partitioning (SVP), evaluates genotype performance based on average performance and stability in a set of environments (Figure 2). The two straight lines, (i) abscissa AEC (vertical) and (ii) ordinate AEC (horizontal) make up this biplot graph. The line in a single direction points to the highest average performance for each trait (Figures 1b, 2b and 3b). The discriminant and representative GGE biplot graph is used to evaluate the tested environments. The size of the vector is important to discriminate the superior genotypes (Figures 1d, 2d and 3d).

#### 3.2.1 Iron content

High Fe contents were identified in G4, G8 and G17 in the SMT, MSH and PIX environments, respectively (Figure 1a). G8> G6> G9> G12 > G7> G10 > G2> G3> G4 were superior to the overall mean of the trials (51.1 mg kg^−1^). G19 and G15 have high phenotypic stability, but low Fe contents. G4, G3 and G7 had specific adaptations for SMT (Figure 1b). G6 was the ideal ideotype and the MSH environment grouped the best genotypes for the trait (Figure 1c). The most discriminating environment was SMT and the most representative was MSH (Figure 1d).

#### 3.2.2 Zinc content

G8, G6, G16, G18 and G11 were genotypes with the highest Zn contents. G11, G6 and G16 had specific adaptability to SMT, PIX and MSH, respectively (Figure 2a). The means of G8> G6> G2> G10 > G11 > G12 > G3> G13 > G4 were higher than the mean of the trials (46.8 mg kg^−1^). Stability was observed for G9, G14, G7 and G1, however, with low Zn contents. G8, G2, G10 and G4 had high Zn contents and stability (Figure 2b). G8 was considered ideal, combining stability and high Zn content (Figure 2c). SMT was the most discriminant and PIX, the most representative (Figure 2d).

#### 3.2.3 Protein content

G16, G8 and G18 were more adapted to PIX, SMT and MSH, respectively (Figure 3a). G8 had a performance higher than the overall mean of the experiments (24.3%) and than all genotypes. G3, G6 and G19 combined phenotypic stability and high protein contents (Figure 3b). G16 and G19 were the most adapted to the SMT (Figure 3c). MSH was the most discriminating and SMT, the most representative for the selection of superior genotypes for high protein contents in the semi-arid region of Piauí (Figure 3d).

## 4 Discussion

The genotypes evaluated in this research showed no genetic variability for GY, Fe and Zn by the F-test. However, this result was expected, since they are inbreeding lines from the Cowpea Breeding Program in the Northeast of Brazil, and have been submitted to several cycles of directional selections (preliminary, intermediate and final trials) with the objective of selecting genotypes with higher yields and nutritional quality, simultaneously (Freire Filho et al., 2011). Furthermore, genealogy of the parents that gave rise to the lines are common among them, favoring inbreeding (see Table 1), making the genetic base restricted. Cowpea is a highly endogamous crop (Kouam et al., 2012), and therefore, genetic variability over successive selection cycles may have reduced (Hedrick; Garcia-Dorado, 2016; Nonaka et al., 2019). Even so, they showed high yield levels for crop, with an average of 1,209.13 kg ha^−1^, being similar to studies reported in the literature (Bastos et al., 2011; Sousa et al., 2019; Cruz et al., 2021).

Two components are described for GEI, "static" and "non-static". The first is made up of characteristics related to location (latitude and longitude) and soil type. The second concerns the climatic variables and the management practices used in the crop (Cullis et al., 2000). The breeder may have two decisions in a breeding program in terms of interaction, either to ignore it or to exploit it during the selection process. When the GEI interaction is ignored, crop-specific adaptations are lost and can compromise yield potential (Yan 2016; Gage et al., 2017). Exploiting GEI allows maximization of trait expression in target population environments, which enables to understand the reaction norms of a genotype in multi-environments (Dias et al., 2018).

The differential effect of environments in this study allows the identification of contrasts between the genotypes, as well as the selection of discriminating and representative environments for the genotypes evaluated in the cowpea VCU trials. Gerrano et al. (2019) in a similar study, identified similar results, where the effect of environment was significant, but without variability for GY. According to Cruz et al. (2014) when the genotype effect is significant in the analysis of variance and it becomes non-existent in the joint ANOVA, the magnitude of the GEI effect is consuming this variability.

Cowpea is a legume with great genetic variability for agronomic, culinary and nutritional traits. It is a promising species for the selection of superior genotypes (Gondwe et al., 2019; Dakora; Belane 2019; Alidu et al., 2020). In the germplasm of Genetic Breeding Program of Embrapa Meio-Norte, genotypes with wide variability for mineral and protein contents in dry grains were identified. This suggests that Brazilian genotypes are promising for genetic biofortification (Carvalho et al., 2012). The environmental effect was significant due to the edaphoclimatic conditions inherent to the cultivation sites (Rocha et al., 2007). The performance of genotypes in multi-environment trials can be altered due to the decrease between genotype and phenotype. This problem hampers the selection of superior genotypes (Abate et al., 2015; Aramendiz et al., 2019).

The effect of GEI is due to physiological and biochemical factors inherent to the genotypes (Cruz et al., 2014). Understanding GEI behavior is very important in breeding programs. This parameter provides important information to assess stability of genotypes across environments, because it can form breeding zones by taking advantage of genotypes with adaptations to specific environments, and indicate genotypes for specific environments to maximize genetic gains (Dias et al., 2018). The GEI detection for nutritional traits reports the response of genotypes in the test environments. Therefore, the edaphoclimatic factors influenced more the response of genotypes in the environments (Santos et al., 2017). The interaction can affect the traits of interest and change the performance of genotypes in the face of environmental changes, making it difficult to recommend new cultivars (Silva et al., 2016; Silva; Santos 2017).

The GGE biplot approach enables several visual interpretations for genotype, environment, and GEI effects compared to the AMMI method (Mekonnen et al., 2022). Through the biplot, crossover interaction can be identified in the multi-environment context, and it is important to be identified when seeking a wide recommendation (Yan et al., 2007; Goa et al., 2022). Principal component analysis (PCA) integrates the GGE biplot approach. The first two principal components (PC) are used in Site Regression (SREG) model, where the first component is more correlated with genotype main effect, and assigns the proportion of the yield that is due to genotype characteristics only. The second component explains the variation in yield in presence of GEI (Yan; Holland 2010; Yan, 2011; Cruz et al., 2020). Therefore, this methodology is based on the singular value decomposition of the two PCs (Yan 2002; Yan; Rajcan, 2002).

The which-won-where biplot is efficient in showing the performance of the best genotypes in their respective environments, and can form target mega-environments for the crop. Genotypes G4 (SMT), G8 (MSH), G17 (PIX) for iron content; G11 (SMT), G16 (MSH), G6 (PIX) for zinc content; and G8 (SMT), G18 (MSH), G16 (PIX) for protein content were the winners in the which-won-where biplot, meaning they had higher mineral contents in their respective environments. The cumulative variation contributed by PC1 and PC2 were 93.84%, 83.63%, 88.72% for Fe, Zn, Prot, respectively. In this regard, size of the vector explains the magnitude of yield in the evaluated environments. Genotypes with smaller vector size within the polygon are considered less responsive to interaction with their test environments (Yan; Rajcan, 2002).

The environments were located in distinct sectors, suggesting that the genotypes had differential response in the environments, evidencing the existence of crossover GEI. In the presence of cross-over interaction it is possible to define target regions for cultivar recommendation by forming mega-environments (Yan; Rajcan, 2002). In addition, it enables the identification of genotypes with specific adaptations, such as G11 for SMT environment for the Zn content trait.

The graph "average" versus "stability" is used for identifying genotypes with high stability and yield. In this biplot, when we have genotypes that are not linked to environments it suggests that they are "unfavorable" for the recommendation, as their performance are inferior to the others (Karimizadeh et al., 2013). Stability is measured by the length of the genotype vector. Those with greater projection imply less stability for the evaluated trait (Yan et al., 2007). The ideotype genotype is identified by the largest projection of the vector, which indicates the point in the center of the concentric circles of the biplot (Figures 1c, 2c and 3c), that use the first two principal components to define the rank of ideotypes (Yan; Rajcan 2002). Furthermore, through this approach, we can identify the behavior of the evaluated environment. Environments with long vectors are able to discriminate genotypes better, and those with short vectors are little discriminating (Yan, 2001). Therefore, Yan (2014) reports that in a genetic breeding program, the selection of the ideal test environment must consider it as discriminating, representative and it must present constant results over the years to select superior genotypes.

The mean Fe content of cowpea genotypes under Piauí semi-arid conditions varied between the evaluated environments. The difference in Fe contents are due to genetic variability and GEI (Silva et al., 2012; Steckling et al., 2017; Silva; Santos 2017; Cardona-Ayala et al., 2021). The existence of three distinct environments showed the presence of complex GEI, which hinders the recommendation of cultivars with broad stability (Eeuwijk et al., 2016). One way to minimize this adversity is to select cowpea genotypes with specific adaptations to the evaluated sites (Putto et al., 2008). G6 was ideal because it is located in the longest vector of the mean environment axis (EAM), where it points to high Fe contents (Horn et al., 2017). The definition of an optimal number of test environments is a relevant factor in genetic breeding programs to increase heritability and the gain with selection, this allows reducing environments with similar responses (Yan et al., 2015).

In a study developed by Silva and Santos (2017) under semi-arid conditions, they identified that the most yielding genotypes are those with greater instability, which makes a broad recommendation difficult. Oliveira et al. (2017) reported that the cultivar BRS Xiquexique was the ideal ideotype, as it presented high Zn contents and stability similar to this study. The genotypes evaluated in this study had 46.87 mg kg^−1^ of Zn in dry grains, a concentration higher than in the study developed by Cardona-Ayala et al. (2021) who found a concentration of 40.9 mg kg^−1^ in cowpea genotypes evaluated in a multi-environment context in Northeastern Colombia.

Genetic variability for protein content in cowpea genotypes was reported under rainfed conditions (Boukar et al., 2011). The variation in protein content between genotypes is related to the genetic factor inherent to the trait (Carvalho et al., 2012). Experimental essays carried by Embrapa Meio-Norte showed positive correlation (0.6354∗) between protein and Fe content in dry grains of cowpea. This indicates the possibility of selection based on a set of traits of interest to the breeder during the stages of the breeding program (Moura et al., 2012). The crude protein contents found in this study confirm the values reported for the crop (Freire-Filho et al., 2012). The evaluated genotypes had higher protein contents than the ones observed by Dias-Barbosa et al. (2021; 22.14%). In that study, GEI can promote changes in behavior of genotypes against environmental variations. Strong GEI for protein content in cowpea lines cultivated in Brazilian semi-arid conditions was found due to environmental conditions (Silva et al., 2016). There is a need to select genotypes with high protein content, especially when one of the strategies is to fight food and nutritional insecurity. It is estimated that more than 239 million individuals are affected by nutritional deficiency, specifically protein calorie (Andrea; Rose 2015).

## 5 Conclusion

The biofortification of cowpea has been developed to increase the mineral and protein contents in grains. The study reported that lines G6 and G8 were promising for iron and zinc, and for high protein contents, in addition to presenting adaptability and stability for the environments of the semiarid zone, Northeast Brazil. The results suggest that the lines have potential for biofortification due to the target minerals of the breeding program and/or use as parents for generation of new breeding populations. Therefore, biofortified crops have a very promising future, as they have the potential to minimize human malnutrition due to lack of micronutrients, especially in underdeveloped and/or developing countries.

## Declarations

### Author contribution statement

Maurício Araújo: Conceived and designed the experiments; Performed the experiments; Analyzed and interpreted the data; Wrote the paper.

Walter Aragão: Analyzed and interpreted the data; Contributed reagents, materials, analysis tools or data.

Samíria Santos, Luiz Antônio Dias: Analyzed and interpreted the data; Wrote the paper.

Thaise Freitas, Keasel Damasceno-Silva: Conceived and designed the experiments; Performed the experiments.

Verônica Saraiva: Contributed reagents, materials, analysis tools or data; Wrote the paper.

Maurisrael Rocha: Conceived and designed the experiments; Performed the experiments; Analyzed and interpreted the data; Contributed reagents, materials, analysis tools or data; Wrote the paper.

### Funding statement

Mauricio dos Santos Araújo was supported by Fundação de Amparo à Pesquisa do Estado do Piauí/ Coordenação de Aperfeiçoamento de Pessoal de Nível Superior -FAPEPI/CAPES [88887.200984/2018–00] and Fundação de Amparo à Pesquisa do Estado de Minas Gerais - FAPEMIG.

Maurisrael de Moura Rocha was supported by HarvestPlus [20.18.01.022.00.00].

### Data availability statement

Data will be made available on request.

### Declaration of interest’s statement

The authors declare no conflict of interest.

### Additional information

No additional information is available for this paper.

## References

[bib1] Abate F., Mekbib F., Dessalegn Y. (2015). Association of different parametric and non-parametric stability models in durum wheat (*Triticum turgidum* Desf.) genotypes. Int. J. Plant Soil Sci..

[bib2] Abreu H.K.A., Ceccon G., Correa A.M., Fachinelli R., Yamamoto E.L.M., Teodoro P.E. (2019). Adaptability and stability of cowpea genotypes via REML/BLUP and GGE biplot. Biosci. J..

[bib3] Alidu M.S., Asante I.K., Mensah H.K. (2020). Evaluation of nutritional and phytochemical variability of cowpea recombinant inbred lines under contrasting soil moisture conditions in the Guinea and Sudan Savanna agro-ecologies. Heliyon.

[bib4] Alvares C.A., Stape J.L., Sentelhas P.C., Moraes G., Leonardo J., Sparovek G. (2013). Köppen’s climate classification map for Brazil. Meteorol. Z..

[bib5] Andrea F., Rose M. (2015). Food insecurity and hunger: a review of FAO's annual report on state of food insecurity in the world. Int. J. Adv. Multidiscip. Res..

[bib6] Aoac - Association Official Analytical Chemists (2005).

[bib7] Aramendiz T.H., Miguel E.C., Carlos C.A. (2019). Adaptation and stability of cowpea (*Vigna unguiculata* (L.) Walp) bean cultivars in the tropical dry forest of Colombia. Aust. J. Crop. Sci..

[bib8] Baraki F., Gebregergis Z., Belay Y., Berhe M., Zibelo H. (2020). Genotype x environment interaction and yield stability analysis of mung bean (*Vigna radiata* (L.) Wilczek) genotypes in Northern Ethiopia. Cogent Food Agric.

[bib9] Bastos E.A., Nascimento S.P., Silva E.M., Freire Filho F.R., Gomide R.L. (2011). Identification of cowpea genotypes for drought tolerance. Rev. Cienc. Agron..

[bib10] Black R.E., Victora C.G., Walker S.P., Bhutta Z.A., Christian P., Onis M., Ezzati M., Grantham-Mcgregor S., Katz J., Martorell R., Uauy R. (2013). Maternal and child undernutrition and overweight in low-income and middleincome countries. Lancet.

[bib11] Bouis H.E., Saltzman A. (2017). Improving nutrition through biofortification: a review of evidence from HarvestPlus, 2003 through 2016. Global Food Secur..

[bib12] Boukar O., Massawe F., Muranaka S., Franco J., Maziya-Dixon B., Singh B., Fatokun C. (2011). Evaluation of cowpea germplasm lines for protein and mineral concentrations in grains. Acta Horticulture.

[bib13] Cakmak I., Kutman U.B. (2017). Agronomic biofortification of cereals with zinc: a review. Eur. J. Soil Sci..

[bib14] Cardona-Ayala C.E., Aramendiz-Tatis H., Camacho M.M.E. (2021). Adaptability and stability for iron and zinc in cowpea by AMMI analysis. Rev. Caatinga.

[bib15] Carvalho A.F.U., Sousa M.N., Farias D.F., Rocha-Bezerra L.C.B., Silva R.M.P., Viana M.P., Gouveia S.T., Sampaio S.S., Sousa M.B., Lima G.P.G., Morais S.M., Barros C.C., Freire-Filho F.R. (2012). Nutritional ranking of 30 Brazilian genotypes of cowpeas including determination of antioxidant capacity and vitamins. J. Food Compos. Anal..

[bib16] Carvalho A.C., Fonsêca P.C.A., Priore S.E., Franceschini S.C.C., Novaes J.F. (2015). Food consumption and nutritional adequacy in Brazilian children: a systematic review. Rev. Paul Pediatr..

[bib17] Conab - Acompanhamento da safra brasileira de grãos Grãos (2021). https://www.conab.gov.br/component/k2/item/download/40129_0ec82309df1a06d3fc177588e37ac0c3.

[bib18] Cruz C.D., Torres R.A.A., Vencovsky R. (1989). An alternative approach to the stability analysis proposed by Silva and Barreto. Rev. Bras. Gent..

[bib19] Cruz C.D., Carneiro P.C.S.E., Regazzi A.J. (2014).

[bib20] Cruz D.P., Geraldo G.A., Vivas M., Entringer G.C., Rocha R.S., Jaeggi M.E.P.C., Gravina L.M., Pereira I.M., Amaral Junior A.T., Ramon M., Oliveira T.R.A., Daher R.F. (2020). Analysis of the phenotypic adaptability and stability of strains of cowpea through the GGE biplot approach. Euphytica.

[bib21] Cruz D.P., Geraldo G.A., Vivas M., Entringer G.C., Souza Y.P., Rocha R.S., Jaeggi M.E.P.C., Albuquerque D.P., Amaral Hunior A.T., Gravina L.M., Rocha M.M., Silva R.K.G. (2021). Combined selection for adaptability, genotypic stability and cowpea yield from mixed models. Ciência Rural..

[bib22] Cullis B.R., Smith A., Hunt C., Gilmour A. (2000). An examination of the efficiency of Australian crop variety evaluation programmes. J. Agric. Sci..

[bib23] Dakora F.D., Belane A.K. (2019). Evaluation of protein and micronutrient levels in edible cowpea (*Vigna unguiculata* L. Walp.) leaves and seeds. Front. Sustain. Food Syst..

[bib24] Dias K.O.G., Gezan S.A., Guimarães C.T., Parentoni S.N., Guimarães P.E.O., Carneiro N.P., Portugual A.F., Bastos E.A., Cardoso M.J., Anoni C.O., Magalhães J.V., Souza J.C., Guimarães M.M.P. (2018). Estimating genotype × environment interaction for and genetic correlations among drought tolerance traits in maize via factor analytic multiplicative mixed models. Crop Breed. Genet..

[bib25] Dias-Barbosa C.Z.M.C., Oliveira D.S.V., Damasceno-Silva K.J.D.S., Moreira-Araújo R.S.R., Rocha M.M. (2021). Selection of cowpea elite lines for iron and zinc biofortification. Curr. Nutr. Food Sci..

[bib26] Eberhart S.A., Russell W.A. (1966). Stability parameters for comparing varieties. Crop Sci..

[bib27] Eeuwijk F.A., Bustos-Korts D.V., Malosetti M. (2016). What should students in plant breeding know about the statistical aspects of genotype x environment interactions?. Crop Sci..

[bib28] Finlay K.W., Wilkinson G.N. (1963). The analysis of adaptation in a plant-breeding programme. Aust. J. Agric. Res..

[bib90] Freire-Filho F.R., Ribeiro V.Q., Rocha M.M., Damasceno-Silva K.J., Nogueira M.S.R., Rodrigues E.V. (2011). Feijão-caupi no Brasil : produção, melhoramento genético, avanços e desafios.

[bib29] Freire-Filho F.R., Ribeiro V.Q., Rocha M.M., Silva K.J.D., Nogueira M.S.R., Rodrigues E.V. (2012).

[bib30] Gage J.L., Jarquin D., Romay C., Lorenz A., Buckler E.S., Kaeppler S., Alkhalifah N. (2017). The effect of artificial selection on phenotypic plasticity in maize. Nat. Commun..

[bib31] Garg M., Sharma N., Sharma S., Kapoor P., Kumar A., Chunduri V., Arora P. (2018). Biofortified crops generated by breeding, agronomy, and transgenic approaches are improving lives of millions of people around the world. Front. Nutr..

[bib32] Gauch H.G. (2006). Statistical analysis of yield trials by AMMI and GGE. Crop Sci..

[bib33] Gerrano A.S., Rensburg W.S.J.V., Kutu F.R. (2019). Agronomic evaluation and identification of potential cowpea (*Vigna unguiculata* L. Walp.) genotypes in South Africa. Acta Agric. Scand B Soil Plant Sci..

[bib34] Goa Y., Mohammed H., Worku W., Urage E. (2022). Genotype by environment interaction and yield stability of cowpea (*Vigna unguiculata* (L.) Walp.) genotypes in moisture limited areas of Southern Ethiopia. Heliyon.

[bib35] Gondwe T.M., Alamu E.O., Mdziniso P., Dixon B.M. (2019). Cowpea (*Vigna unguiculata* (L.) Walp.) for food security: an evaluation of end-user traits of improved varieties in Swaziland. Sci. Rep..

[bib36] Harvestplus (2021). https://www.harvestplus.org/biofortification-nutrition-revolution-now.

[bib37] Hedrick P.W., Garcia-Dorado A. (2016). Understanding inbreeding depression, purging, and genetic rescue. Trends Ecol. Evol..

[bib38] Hohenberger J., Ray K., Meyer K. (2012). The biology and chemistry of high-valent iron–oxo and iron–nitrido complexes. Nat. Commun..

[bib39] Horn L., Shimelis H., Sarsu F., Mwadzingeni L., Laing D.M. (2017). Genotype-by-environment interaction for grain yield among novel cowpea (*Vigna unguiculata* L.) selections derived by gamma irradiation. Crops J.

[bib40] Issoufa B.B., Ibrahim A., Abaidoo R.C. (2020). Agronomic and economic benefits of integrated nutrient management options for cowpea production. Exp. Agric..

[bib41] Karimizadeh R., Mohammadi M., Sabaghni N., Mahmoodi A.A., Roustami B., Seyyedi F., Akbari F. (2013). GGE biplot analysis of yield stability in multienvironment trials of lentil genotypes under rainfed condition. Not. Sci. Biol..

[bib42] Khang D.T., Dung T.N., Elzaawely A.A., Xuan T.D. (2016). Phenolic profiles and antioxidant activity of germinated legumes. Foods.

[bib43] Kihara J., Bolo P., Kinyua M., Piikki K. (2020). Micronutrient deficiencies in african soils and the human nutritional nexus: opportunities with staple crops. Environ. Geochem. Health.

[bib44] Koop A.H., Mousa O.Y., Pham L.Y.E., Corral-Hurtado J.E., Pugpapong S., Keaveny A.P. (2018). An argument for vitamin D, A, and zinc monitoring in cirrhosis. Ann. Hepatol..

[bib45] Kouam E.B., Pasquet R.S., Campagne P., Tignegre J.B., Thoen K., Gaudin R., Ouedraogo J.T., Salifu A.B., Muluvi G.M., Gepts P. (2012). Genetic structure and mating system of wild cowpea populations in West Africa. BMC Plant Biol..

[bib46] Lin C.S., Binns M.R. (1988). A superiority measure of cultivar performance for cultivar x location data. Can. J. Plant Sci..

[bib47] Lonergan Z.R., Skaar E.P. (2019). Nutrient zinc at the host–pathogen interface. Trends Biochem. Sci..

[bib48] López-Morales D., Cruz-Lázaro E., Sánchez-Chávez E., Preciado-Rangel P., Márquez-Quiroz C., Osorio-Osorio R. (2020). Impact of agronomic biofortification with zinc on the nutrient content, bioactive compounds, and antioxidant capacity of cowpea bean (*Vigna unguiculata* L. Walpers). Agronomy.

[bib49] Mariotti F., Tomé D., Mirand P.P. (2008). Converting nitrogen into protein - beyond 6.25 and Jones' factors converting nitrogen into protein. Crit. Rev. Food Sci. Nutr..

[bib50] Mekonnen T.W., Mekbib F., Amsalu B., Gedil M., Labuschagne M. (2022). Genotype by environment interaction and grain yield stability of drought tolerant cowpea landraces in Ethiopia. Euphytica.

[bib51] Mendiburu F. (2021). https://CRAN.R-project.org/package=agricolae.

[bib52] Mohammed S.T., Amsalu W.B. (2018). Genotype by environment interaction and stability analysis of cowpea [*Vigna unguiculata* (L.) Walp.] genotypes for yield in Ethiopia. Rev..

[bib53] Moreira-Araújo R.S.R., Sampaio G.R., Soares R.A.M., Silva C.P., Arêas J.A.G. (2017). Identification and quantification of antioxidant compounds in cowpea. Rev. Cienc. Agron..

[bib54] Moura J.O., Rocha M.M., Gomes R.L.F., Freire-Filho F.R., Damasceno-Silva K.J., Ribeiro V.Q. (2012). Path analysis of iron and zinc contents and others traits in cowpea. Crop Breed. Appl. Biotechnol..

[bib55] Naik S.M., Raman A.K., Nagamallika M., Venkateshwarlu C., Singh S.P., Kumar S., Singh S.K., Ahmed T., Das S.P., Prasad K., Izhar T., Mandal N.P., Singh N.K., Yadav S., Reinke R., Swamy B.P.M., Virk P., Kumar A. (2020). Genotype × environment interactions for grain iron and zinc content in rice. J. Sci. Food Agric. J. Sci..

[bib56] Nonaka E., Sirén J., Somervuo P., Ruokolainen L., Ovaskainen O., Hanski I. (2019). Scaling up the effects of inbreeding depression from individuals to metapopulations. J. Anim. Ecol..

[bib57] Okoth J.K., Ochola A.S., Gikonyo N.K., Makokha A. (2017). Development of a nutrient-dense complementary food using amaranth-sorghum grains. Food Sci. Nutr..

[bib58] Oliveira D.S.V., Franco L.J.D., Menezes-Júnior J.A.N., Damasceno-Silva K.J., Rocha M.M., Neves A.C., Sousa F.M. (2017). Adaptability and tability of the zinc density in cowpea genotypes through GGE-biplot method. Rev. Cienc. Agron..

[bib59] Olivoto T., Lúcio A.D. (2020). Metan: an R package for multi-environment trial analysis. Methods Ecol. Evol..

[bib60] Ongom P.O., Fatokun C., Togola A., Oyebode O.G., Ahmad M.S., Jockson I.D., Bala G., Boukar O. (2021). Genetic worth of multiple sets of cowpea breeding lines destined for advanced yield testing. Euphytica.

[bib61] Putto W., Patanothai A., Jogloy S., Hoogenboom G. (2008). Determination of mega-environments for peanut breeding using the CSM-CROPGRO-peanut model. Crop Sci..

[bib62] R Core Team (2022). http://www.R-project.org/.

[bib63] Rocha M.D.M., Freire-Filho F.R., Ribeiro V.Q., Carvalho H.W.L., Belarmino-Filho J., Raposo J.A.A., Alcântara J.P., Ramos S.R.R., Machado C. (2007). Adaptabilidade e estabilidade produtiva de genótipos de feijão-caupi de porte semi-ereto na região Nordeste do Brasil. Pesqui. Agropecu. Bras..

[bib64] Roohani N., Hurrell R., Kelishadi R., Schulin R. (2013). Zinc and its importance for human health: an integrative review. Res. J. Med. Sci..

[bib65] Rouault T.A. (2015). Iron-sulfur proteins hiding in plain sight. Nat. Chem. Biol..

[bib66] Santos A., Ceccon G., Rodrigues E.V., Teodoro P.E., Correa A.M., Torres F.E., Alvarez R.C.F. (2017). Selection of cowpea genotypes for Mato Grosso do Sul via GGE biplot and linear regression. J. Biosci..

[bib67] Santos S.P., Damasceno-Silva K.J., Aragão W.F.L., Araújo M.S., Rocha M.M. (2020). Genetic control of traits related to maturity in cowpea. Crop Breed. Appl. Biotechnol..

[bib68] Scott A.J., Knott M. (1974). A cluster analysis method for grouping means in the analysis of variance. Biometrics.

[bib69] Silva D.O.M., Santos C.A.F. (2017). Adaptability and stability parameters of iron and zinc concentrations and grain yield in cowpea lines in the Brazilian semiarid region. Crop Sci..

[bib70] Silva C.A., Abreu A.Â.F.B., Ramalho M.A.P., Corrêa A.D. (2012). Interaction genotype by season and its influence on the identification of beans with high content of zinc and iron. Bragantia.

[bib71] Silva D.O.M., Santos C.A.F., Boiteux L.S. (2016). Adaptability and stability parameters of total seed yield and protein content in cowpea (*Vigna unguiculata*) genotypes subjected to semi-arid conditions. Aust. J. Crop. Sci..

[bib72] Sousa T.J.F., Rocha M.M., Damasceno-Silva K.J., Bertini C.H.C.M., Silveira L.M., Sousa R.R., Sousa J.L.M. (2019). Simultaneous selection for yield, adaptability, and genotypic stability in immature cowpea using REML/BLUP. Pesqui. Agropecu. Bras..

[bib73] Steckling M.S., Ribeiro N.D., Arns F.D., Mezzomo H.C., Possobom M.T.D.F. (2017). Genetic diversity and selection of common bean lines based on technological quality and biofortification. Genet. Mol. Res..

[bib74] Tardy A.L., Pouteau E., Marquez D., Yilmaz C., Scholey A. (2020). Vitamins and minerals for energy, fatigue and cognition: a narrative review of the biochemical and clinical evidence. Nutrients.

[bib75] Ullah A., Al-Sadi A.M., Al-Subhi A.M., Farooq M. (2020). Characterization of chickpea genotypes of Pakistani origin for genetic diversity and zinc grain biofortification. J. Sci. Food Agric..

[bib76] Weng Y., Qin J., Eaton S., Yang Y., Ravelombala W.S., Shi A. (2019). Evaluation of seed protein content in USDA cowpea germplasm. Hortscience.

[bib77] Who – World Health Organization (2022). https://www.who.int/health-topics/micronutrients#tab=tab_1.

[bib78] Yan W. (2001). GGE biplot: a windows application for graphical analysis of multienvironment trial data and other types of two-way data. Agron. J..

[bib79] Yan W. (2002). Singular-value partitioning in biplot analysis of multi environment trial data. Agron. J..

[bib80] Yan W. (2011). GGE biplot vs. AMMI graphs for genotype-by-environment data analysis. J. Indian Soc. Agric. Stat..

[bib81] Yan W. (2014).

[bib82] Yan W. (2016). Analysis and handling of GxE in a practical breeding program. Crop Sci..

[bib83] Yan W., Holland J.B. (2010). A heritability-adjusted GGE biplot for test environment evaluation. Euphytica.

[bib84] Yan W., Rajcan I. (2002). Biplot Analysis of test sites and trait relations of soybean in Ontario. Crop Sci..

[bib85] Yan W., Tinker A. (2006). Biplot analysis of multi environment trial data: principles and applications. Can. J. Plant Sci..

[bib86] Yan W., Hunt L.A., Sheng Q., Szlavnics Z. (2000). Cultivar evaluation and mega-environment investigation based on the GGE biplot. Crop Sci..

[bib87] Yan W., Kang M.S., Ma B., Woods S., Cornelius P.L. (2007). GGE biplot vs. AMMI analysis of genotype-by-environment data. Crop Sci..

[bib88] Yan W., Frégeau-REID J., Martin R., Pageau D., Mitchell-Fetch J.W. (2015). How many test locations and replications are needed in crop variety trials in a target region?. Euphytica.

